# Saving the Last West African Giraffe Population: A Review of Its Conservation Status and Management

**DOI:** 10.3390/ani14050702

**Published:** 2024-02-23

**Authors:** Kateřina Gašparová, Julian Fennessy, Abdoul Razack Moussa Zabeirou, Ali Laouel Abagana, Thomas Rabeil, Karolína Brandlová

**Affiliations:** 1 Faculty of Tropical AgriSciences, Czech University of Life Sciences Prague, Kamýcká 129, 165 00 Prague, Czech Republicmoussa_zabeirou@ftz.czu.cz (A.R.M.Z.); 2 Giraffe Conservation Foundation, Windhoek 10009, Namibia; julian@giraffeconservation.org; 3 School of Biology and Environmental Science, University College Dublin, D04 V1W8 Dublin, Ireland; 4 Project Sustainable Management of Biodiversity, Ministry of Environment and Sustainable Development, Niamey 920001, Niger; 5 Wild Africa Conservation, Kouara Kano, BP32, Niamey 920001, Niger

**Keywords:** community engagement, conservation translocation, *Giraffa camelopardalis peralta*, Niger, human–wildlife conflict, West African giraffe, wildlife survey

## Abstract

**Simple Summary:**

This review focuses on the West African giraffe and summarizes their past and present conservation management activities. It evaluates their impact to advise and prioritize future conservation actions moving forward. This review covers monitoring and annual censuses, local community engagement, habitat use, and translocation. Recommendations for the long-term conservation of the West African giraffe are provided as a summary.

**Abstract:**

The West African giraffe (*Giraffa camelopardalis peralta*) was historically spread across much of the Sudano-Sahelian zone but is now restricted to Niger. Several factors resulted in their dramatic decline during the late 20th century. In 1996, only 49 individuals remained, concentrated in the ‘Giraffe Zone’. Conservation activities implemented by the Government of Niger, supported by local communities and NGOs, facilitated their population numbers to increase. This review summarizes past and present conservation activities and evaluates their impact to advise and prioritize future conservation actions for the West African giraffe. The long-term conservation of the West African giraffe is highly dependent on the local communities who live alongside them, as well as supplementary support from local and international partners. Recent conservation initiatives range from community-based monitoring to the fitting of GPS satellite tags to better understand their habitat use, spatial movements to expansion areas, and environmental education to the establishment of the first satellite population of West African giraffe in Gadabedji Biosphere Reserve, the latter serving as a flagship for the future restoration of large mammal populations in West Africa. The integration of modern technologies and methods will hopefully provide better-quality data, improved spatial analyses, and greater understanding of giraffe ecology to inform the long-term management of West African giraffe.

## 1. Introduction

The West African savanna and Sahel region, historically hosting diverse and abundant wildlife species, now faces increasing pressure from an escalating human population and insecurity, which may lead to increased habitat loss and degradation, poaching, and other associated impacts [1]. Many large- and medium-sized mammal taxa endemic to the region have dramatically declined in the last century [2], e.g., West African lion (*Panthera leo leo*) [3], Derby eland (*Tragelaphus derbianus*) [4,5], African savanna and forest elephant (*Loxodonta africana* and *L. cyclotis*) [6,7], and cheetah (*Acinonyx jubatus*) [8]. The main drivers of such declines are similar for each species, including the degradation of habitat by fragmentation, illegal hunting (poaching), armed (civil) war, competition with livestock, increasing agricultural land, and climate change [9,10]. The majority of West Africa’s megafauna does not persist outside protected areas [1,11]. One of the few exceptions in the region is the West African giraffe (*Giraffa camelopardalis peralta*) [12,13], a subspecies of the northern giraffe (*G. camelopardalis*) [14,15,16], which rebounded from the brink of extinction in the mid-1990s [17]. Found almost exclusively outside formally protected areas, the West African giraffe population steadily increased by over 1200% in the past three and a half decades, resulting in its downlisting from Endangered (2008) to Vulnerable (2018) on the IUCN Red List [18].

In the late 19th century, the West African giraffe geographical distribution ranged from Senegal in the west, through Mauritania, Gambia, Mali, and Niger, to western Nigeria [19]. A number of geographical barriers, e.g., Niger River, Upper Guinea Forest, etc., likely prevented their distribution across other West and Central African countries; however, by the beginning of the 20th century, the West African giraffe was extirpated from most of its former range [13,19,20]. In the late 1960s, only a few West African giraffe persisted in Mali, Niger, and Senegal [19].

In Niger, the West African giraffe population was extirpated in the Gadabedji Biosphere Reserve (GBR) in the 1970s because of severe drought, which led to illegal hunting [21]. Similar local extirpations occurred in the Ayorou and Tanout area between Agadez and Zinder in the mid-1980s [22]. The first coordinated effort in Niger to curb illegal hunting was initiated in the early 1980s, yet the demise of the West African giraffe continued [23]. In 1996, the remaining population was estimated at only 49 giraffes, and they were geographically restricted to an area of arid Sahelian scrubland in the Kouré area, commonly referred to as the ‘Giraffe Zone’. Occasionally, individuals or small herds seasonally migrated from the core range west towards the Mali border (Ouallam) and east towards Gaya close to the Nigerian border [21,22,24].

In 2008, the first ever national strategy for giraffe conservation was developed together with the population viability assessment (PVA) [25], highlighting the need of establishing additional giraffe populations, understanding the habitat use of the giraffe, working with communities, and preventing habitat loss and conflicts related to the expansion of agriculture in the area. Similar points were raised again in the second West African giraffe strategy in 2016 in line with the IUCN Strategy planning guidelines [26].

**Figure 1:**
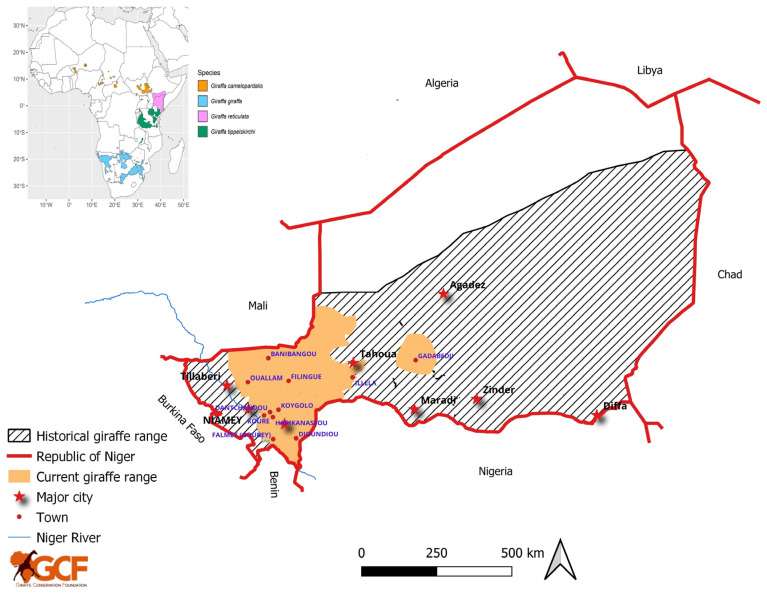
Historical and current distribution of West African giraffe in Niger. Inset shows current distribution of all four giraffe species across their range in Africa.

Today, the West African giraffe predominantly inhabits the ‘Giraffe Zone’, north of the Niger River around Kouré, starting ~60 km southeast of the capital, Niamey [20,24], and Gadabedji Biosphere Reserve (BR), which is located in the Sahelian grasslands of central Niger (Figure 1).

In this review, we aimed to summarize past and present conservation management activities for the West African giraffe in Niger and evaluate their impact to advise and prioritize future conservation actions.

## 2. Methods

We undertook an online review of all the relevant scientific publications on Web of Science and Google Scholar, in addition to the available grey literature (project reports, national action plans, conservation strategies, and theses), which highlighted and/or described the ecology and conservation activities of the West African giraffe. During the review, the following keywords were used to ascertain publications related to the West African giraffe: girafe, giraffe, *Giraffa camelopardalis peralta*, Giraffe Zone, Niger, Girafe d’Afrique de l’Ouest, coexistence. Additionally, we compiled an updated profile of the current activities around the conservation and management of the West African giraffe.

## 3. Results

After the online keyword review of Web of Science and Google Scholar, only 12 scientific papers and 31 grey literature articles referring to the West African giraffe were found. The topics of the papers included conservation activities and census monitoring (16), conservation (13), ecology (5), threats (4), demography (2), human–wildlife coexistence (2), and behavior (1).

### 3.1. Monitoring and Annual Census

Understanding the dynamics of a population is critical for sound conservation planning and management, especially of threatened species [27]. For surveys, regular repeatability and replicability allows for a more accurate assessment of species’ demographic parameters [28]. When accompanied with individual attributed population data, e.g., age and sex, one can predict population growth trajectory. Importantly, understanding changes in population size can serve as a proxy for the effectiveness of conservation actions and management practices.

Giraffe’s individually unique pelage pattern remains largely unchanged throughout their life and, as such, is a valuable tool for monitoring, e.g., [17,29,30,31,32]. The first individual identification census of the West African giraffe in Niger was conducted in 1995 with support from the U.S. Peace Corps, resulting in a population estimate of 49 individuals [33]. Between 1996 and 1999, individual giraffe photographic albums were established through regular surveys under the framework of the “Projet d’Utilisation des Resources Naturelles de la region de Kouré et Dallol Bosso Nord (PURNKO)”. No surveys were then undertaken until 2005 when J.P. Suraud, working with the Association for Saving the Giraffes of Niger (ASGN), re-initiated efforts [34,35]. Since then, an annual census in Niger, except for 2001 and 2003, was coordinated by the Government of Niger with support from local community and international NGO partners [17,21,36,37,38]. Over the 24 years of surveying, three different methods have been used: (1) ground strip transects (2002), (2) total aerial count (2004), and, predominantly, (3) individual photo identification (1996 to 1998 and 2005 onwards).

Both the ground strip transects survey (2002) and the aerial survey (2004) reported more bias than the ground surveys [35]. The ground strip transect method (10 days, 57 transects, 27 km long) led to more biases, such as the small number of observers on motorcycles, lack of individual identification data, double-counting errors, and transect placement [35]. In contrast, the aerial survey was time–cost-effective and covered the whole core ‘Giraffe Zone’, deploying six people in three ultralight aircrafts undertaking half-day linear transects (1 km apart). However, the results were limited as a standalone assessment, and there was a lack of comparable data, e.g., no individual identification data were recorded, nor age–sex class information, coupled with a large underestimation of the population due to transect widths and limited visibility [22,35]. The long-term vehicle-based surveys provided the most exhaustive and least biased results, despite a level of reduced visibility and accessibility. It proved to be the most reliable method when giraffe numbers were low, and distribution was relatively restricted; hence, the timing of the survey was critical. However, the ground surveys were not comparatively time–cost-effective, especially as more people were involved over an increased time period, and post-survey analysis was time consuming.

The annual survey systematically took place during the rainy season, as giraffes are concentrated on the Kouré and Fandou plateaus. All the giraffes observed were identified, and subsequently they were updated on or added (if new) to the West African giraffe database and initially categorized into one of three age classes: juvenile (6–18 months), subadult (18 months—4 years), and adult (>4 years) [30]. In 2005, a fourth age class (calf—<6 months) was added to further refine the population structure [35]. Each survey deployed more than 20 people over approximately 45 days, daily searching the area in vehicles until no new giraffes were observed [35,39]. Most recently, the Giraffe Conservation Foundation (GCF) initiated a hot–dry season census to complement the annual survey to assess the most efficient population estimation method for the population [40]. As the population increased, individual identification became more challenging due to their expanded range, and the probability of individual misidentification was multiplying. Unknown, missing, double-counting, and/or deceased animals have likely resulted in higher annual population estimates in recent years. Therefore, to best counter these survey challenges in the future, there is a need to assess and develop relatively time-, cost-effective, and reliable survey and analysis methods to better estimate population numbers [40,41].

**Figure 2:**
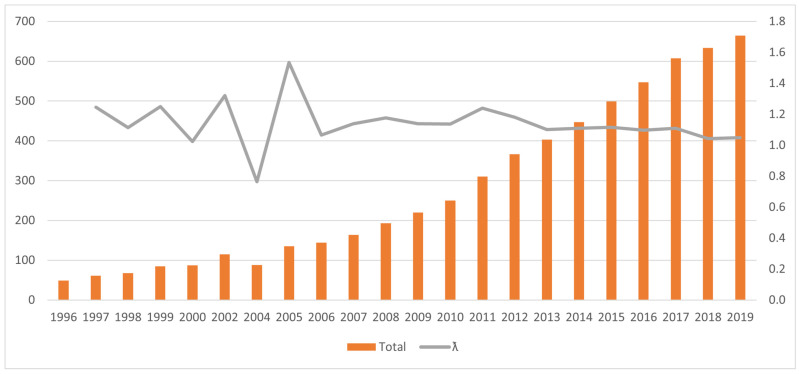
The West African giraffe population estimates in Niger from 1996 to 2019. Note: individual photo identification was the most used survey method, except in 2002 when a ground strip transect was used, and in 2004 when an aerial census was conducted. Left Y axis: the total number of individual giraffes counted each year. Right Y axis: ƛ represents the finite rate of growth calculated as (N + 1)/N), where N represents total number of individual giraffes counted in a specific year.

Since there was a low number of individuals in 1996, namely 49, the population consistently increased. It reached the highest mean annual growth rate (19%) between 1996 and 1999, immediately following the implementation of the first conservation actions. Until 2005, the survey methods were inconsistent, and it was therefore not possible to accurately evaluate the annual population growth rate. In the following 10 years, the average generation length of a giraffe [42], the mean annual growth rate was 14%, decreasing to 7% between 2016 and 2019 (Figure 2). The population growth of the West African giraffe has been truly remarkable, yet the current growth trend is decreasing, a likely indicator of the population reaching carrying capacity in the ‘Giraffe Zone’.

### 3.2. Local Community Engagement

During the mid- to late-1990s, the Government of Niger, alongside local communities with support from WWF, undertook the first targeted efforts to save the last West African giraffe [43]. In September 1996, the PURNKO program, as well as initiating scientific studies and monitoring the giraffe, launched several community projects, which led to the local cessation of poaching. Based on the principles of sustainable natural resources management, PURNKO integrated giraffe conservation within the protection of the “tiger bush” or “brousse tigrée” habitat in the ‘Giraffe Zone’ and the neighboring ephemeral river system. Among other activities, ecotourism opportunities were initiated alongside various development projects, including soil restoration, forestry, land use management for pastoralist and farming communities, and a microcredit program for women. Following the project end of PURNKO in mid-1999, the future development of those conservation actions was uncertain [34].

As an alternative livelihood opportunity, the Association pour la Valorisation de l´Ecotourisme au Niger (AVEN) was established in 2000 as a local ecotourism initiative to visit the West African giraffe. AVEN and its guides operate in the ‘Giraffe Zone’ and primarily cooperated with local and international tourist operators to facilitate tourist visits to observe the giraffe, as well as support local education and awareness around giraffes. In the late 2000s, a tourist information center and base for AVEN was established in Kouré with the support of international donor funding. In recent years, regular tourists visited during the peak season; however, tourism totally ceased because of the COVID-19 pandemic and a terrorist attack in August 2020 near Kouré, where six French humanitarians, their driver, and the President of AVEN were killed [44]. Over the past two decades, AVEN has played a critical role in the annual giraffe census and ongoing monitoring of the giraffe in the ‘Giraffe Zone’, as well as increasing local awareness and education.

In 2001, the L’Association de sauvegarde des girafes du Niger (ASGN) was established with the support of Bioparc de Doué-la-Fontaine and other French zoos and organizations [45]. In the past two decades, the ASGN has helped to protect the West African giraffe through targeted development programs across the ‘Giraffe Zone’ and neighboring areas. Such support includes improving local peoples’ living conditions through access to water, microcredits, education, awareness, monitoring, and more. With an increasing giraffe range, the ASGN more recently expanded their field operations into the Dingazi Region, north of the ‘Giraffe Zone’. Importantly, the positive efforts of the ASGN have been driven from a community-based conservation development approach with a direct link to the long-term management of the West African giraffe.

The positive coexistence of local communities with the West African giraffe has resulted from direct local development and humanitarian assistance. Such support has inevitably been a key factor in curbing giraffe poaching, with relatively few cases reported since 2000 [34,35]. These included five vagrant giraffes from Niger which were poached in Mali in 2000 and two in Nigeria in 2007, as well as a few individual poaching cases in Niger ([17], I. Ciofolo pers. comm.). The coexistence of local people and giraffes is neither unique nor uniform throughout Africa, similarly, occurring in Kenya, Namibia, and Tanzania, for example [13]. However, the relatively harmonious coexistence in the first decade after the establishment of illegal hunting pressure in Niger was a laudable effort spearheaded by the government and NGOs [34,43,46,47].

Over the past few decades, the increasing population numbers and range of both humans and giraffes in Niger has led to growing human–giraffe conflict [47,48]. Daytime crop raiding by giraffes, e.g., cowpeas *Vigna unguiculata*, has been further exacerbated by the night time raiding of mangoes *Mangifera indica* [45,47,48,49,50,51,52,53,54]. In retaliation, some local community members have threatened giraffes by chasing them with modified weapons [51], with at least one giraffe being killed [43]. To minimize these conflicts, many local people have fenced their mango trees, but the fields are too large, and giraffes prefer foraging trees which are located within and around them ([33], O. Idrissa, pers. comm.). Despite a high tolerance towards giraffes, conflict will likely increase as both populations grow and competition for resources rises [54]. The carrying capacity of giraffes in the ‘Giraffe Zone’ is hard to assess; however, as the food resources decrease, giraffes are seeking more favorable habitats further away from the core ‘Giraffe Zone’, with greater food availability and fewer human disturbances ([35], A. Zabeirou pers. Comm.). Aside from natural expansion, human–giraffe conflict may likely only be alleviated by augmentation and the creation of new satellite populations of the West African giraffe in their former range across Niger and/or regionally.

### 3.3. Habitat Use

Wildlife population distribution patterns result from individual behavioral processes and are often associated with plant phenology, forage availability, reduction in competition, predator avoidance, and/or avoidance of harsh weather conditions, to name but a few [55,56,57,58,59,60]. Moreover, and in the context of the West African giraffe, spatial behavior may be influenced by increasing human population and associated disturbances [61].

The remote tracking of large mammals allows for a detailed understanding of individual’s movements, revealing daily and seasonal space-use patterns. The remote tracking of large mammals has become increasingly accessible, with the first giraffe being tracked—an Angolan giraffe *G. giraffa angolensis*—in 2002 using a GPS satellite unit in Namibia [31]. The first tracking of the West African giraffe using GPS satellite was subsequently undertaken in 2010 in Niger [35]. Eight collars designed to sit around the base of the giraffe neck were fitted to females and set to send hourly positions. Unfortunately, due to design limitations, they were removed after three months of monitoring because of the irritations and superficial injuries they caused to the giraffes [35]. Importantly, all giraffes recovered well, and all individuals subsequently reproduced (A. Zabeirou pers. comm.).

In a predator-free environment, the population distribution of a prey species is expected to be driven mostly by seasonal forage availability, disease, intra-specific competition, and human disturbances. Suraud [35] observed two separately and seasonally used core home range (HR) areas of the West African giraffe. During the rainy season, almost all (91%) giraffes resided in the Kouré area, where the ‘tiger bush’ is highly productive. However, during the dry season, 85% of the giraffe population were observed in the north Dallol Bosso area, where the ephemeral riparian environments around Harikanassou and Dallol Bosso are relatively abundant in preferred giraffe forage: *Faidherbia albida*, *Combretum glutinosum*, *Balanites aegyptiaca*, and *Prosopis africana* [24,30,35]. Transition movements were observed late in the dry season (late April to late May) when the giraffe moved to the Kouré area, coinciding with the leaf flush of the tiger bush before the rains come, and at the end of the rainy season (November), when giraffe moved to the north Dallol Bosso area to take advantage of the abundant reverse phenology of *F. albida* during the dry season [30,35].

The first HR sizes for the West African giraffe were calculated using both the Kernel Density Estimator (KDE) and Minimum Convex Polygon (MCP), further highlighting the variance between the seasons. The West African giraffe average HR was estimated at 398 km^2^ (KDE)/47 km^2^ (MCP) during the rainy season and 507 km^2^ (KDE)/91 km^2^ (MCP) during the dry season [24,35]. It was hypothesized that giraffes avoided areas of increased human presence which were higher in north Dallol Bosso because of increased water availability and better-quality soils for agriculture. Despite the short-term data collection achieved by the initial GPS satellite collars, the preliminary results highlighted variances between giraffe diurnal and nocturnal habitat use. Their diurnal selection appears to be shaped by human disturbances, and giraffes moved closer to (and in) the villages at night, especially when a water point was close and the tree density was high [37]. Some giraffes appeared to be non-resident, occasionally migrating west/northwest towards the Mali border (Ouallam district), and others east towards the Nigerian border (Gaya district) [22,24,60,61,62]. Seeking new habitats and the long-distance movement towards (and into) Mali and Nigeria associated with this have and will likely continue to present a poaching threat [22,43,60].

In 2018–19, 19 (15 females and 4 males) giraffes were fitted with GPS solar-powered satellite tags as part of the GCF’s collaborative Africa-wide Twiga Tracker Initiative to obtain updated knowledge of their habitat utilization and spatial ecology resulting from an increasing population and range extension of the West African giraffe. This activity was a key output in the implementation of the Government of Niger’s second National Giraffe Conservation Strategy [26]. The longevity and function of the units varied between individuals ranging from 2 to 28 months, with units fitted to males all stopping transmission prematurely, likely because of damage caused to the units during necking behavior. The data obtained were invaluable in better understanding current giraffe movements and habitat use, confirming previous seasonal migrations reported and highlighting range expansions [61,63,64,65].

### 3.4. Translocation

Translocations are considered among the most powerful tools in biodiversity conservation, with the aim of maintaining or reinvigorating biodiversity and ecosystem function [66]. Undertaking pre- and post-feasibility analysis to assess such actions and the subsequent success of any translocation is critical, although unfortunately not common.

The establishment of a new ‘satellite’ population(s) in Niger was assessed and identified as a feasible and desirable measure for the long-term viability of the West African giraffe, and a key objective in both the first and second National Giraffe Conservation Strategy [25,26]. From a conservation perspective, as numbers of populations increase, the demographic and environmental stochasticity risks reduce.

An initial feasibility study to assess giraffe translocation potentials within Niger was presented and discussed during the second National Strategy workshop in 2014 [26]. The methodology for site selection included GIS analysis and the desktop review of West African giraffe historical range and other giraffe translocation successes, combined with detailed discussions with the Nigerian authorities and a participatory survey of 20 experts who prioritized key translocation elements and the most appropriate sites for release. The assessment proposed three potential translocation sites in Niger: (1) GBR in the central areas, (2) Tadrès Total Reserve in the central north, and (3) Park W on the southwestern border. Each of these sites were objectively analyzed using criteria that influenced the distribution and dynamic of the population, e.g., distance to roads, land use, percentage tree cover, human density, distance to river system, vegetation index NDVI, precipitation, and climate change [26]. Park W was selected as the most suitable for translocation but was rejected for political, security, and strategic reasons with the aim of keeping giraffes in the country and not too close to the neigboring Benin and Burkina Faso within the open Park W ecosystem. Additionally, the potential risk of lion predation coupled with the limited evidence of the West African giraffe historical presence south of the Niger River was highlighted. Whilst identified as a potential site, the GBR was not initially selected as the primary location because of the historical poaching in the area and high latitude linked to the risk of long-term climate change. However, following discussions at the second National Strategy workshop, it was subsequently recommended that the first translocation and establishment of a satellite West African giraffe population should be in GBR as local conditions had become more favorable [67,68,69].

Over more than a year, several meetings, workshops, and field visits were undertaken to assess the feasibility and plan the practicalities of any potential translocation [70,71]. During the planning, aspects such as the siting of the boma (holding pen) in the ‘Giraffe Zone’ close to a secondary transport route and being easily accessible for the translocation truck were taken into account, and the route from the boma to GBR was checked for road condition, powerlines, and other potential obstacles. In GBR, the team assessed forage availability and abundance, access to water, security, potential release sites, and more, and all agreed that GBR provided a good long-term habitat for giraffes [70]. Once approved by the Government of Niger, the necessary equipment was obtained, a field recovery trailer (chariot) and translocation truck were assembled, and the bomas were built.

In November 2018, the first ever translocation of the West African giraffe was undertaken by GCF in support of the Government of Niger, with assistance from local and international partners. Eight subadult giraffes (five females and three males) were individually immobilized and captured in the ‘Giraffe Zone’. After habituating for three weeks in the boma, they were transported ~800 km east to GBR, approximately 50 years after their local extinction [72]. Following their release, ongoing monitoring of the giraffe’s movements, social behavior, and forage preferences has been undertaken ([72], R. Zabeirou pers. comm.). Local community Tuareg ecoguards were recruited by GCF and trained in Cybertracker and Garmin InReach technology and worked alongside the local GBR rangers to monitor the giraffes as well as raise local awareness among communities. Additionally, in 2022, an additional four giraffes were translocated from the ‘Giraffe Zone’ to the GBR [J. Fennessy pers. comm.]. Since the translocations, all giraffes have successfully coexisted with the local community, including sharing water points with livestock. The population has expanded, with five calves born, showing early signs of success in the first five years after the initial translocation. Occasionally, the giraffes undertake forays out of the reserve, but with the support of the ecoguards, they are guided back to a stress-free environment. Interestingly, the preferred forages are *Ziziphus mauritania*, *F. albida*, *Vachellia seyal*, and *V. raddiana* [73,74,75], which are similar forage species to those eaten by giraffes in the ‘Giraffe Zone’ and across their range throughout Africa [44]. Long-term post-translocation monitoring has and will continue to be crucial to evaluate their translocation success and advise on future translocations to GBR and other potential sites in the country or regionally.

## 4. Discussion

The conservation success of the rebounding West African giraffe population is a direct result of good policy, governance, and community-based conservation activities. However, the valuable community support observed to date may in time become compromised by the increasing numbers of not only giraffes, but also human and livestock populations. This, in turn, may lead to increased human–giraffe conflict. In understanding the increasing giraffe population and their relationships with the available resources, growing human settlements, local infrastructure development, etc., the continued use of GPS satellite technology, combined with targeted ongoing monitoring and adapted surveys, will help to inform better decision making. Additionally, to decrease the risk of stochastic events such as droughts, disease, insecurity, etc., which are currently a risk in the majority ‘Giraffe Zone’ population, further conservation translocations should be considered to other approved areas in the country and regionally, so that ‘all eggs are not in the one basket’.

Long-term annual surveys of the West African giraffe population using ground counts methods have provided the best comparable data for assessing their growth and guiding conservation management [17,32,41]. However, with increasing giraffe numbers, coupled with human population and livestock growth in the ‘Giraffe Zone’, their range expansion [76] and insecurity in the region will be challenging to implement population-wide targeted monitoring. The capture–mark–recapture (CMR) methods have been successfully applied to other large giraffe populations across their range to estimate density and abundance, whilst also monitoring age- and sex-specific survival rates and other life history parameters, all valuable for targeted conservation management, e.g., [30,77,78]. Where the range size and/or security situation does not allow for direct observations, data from CMR surveys in the most accessible areas can be used as a proxy for the validation of the effectiveness of other survey techniques (namely aerial surveys) and provide a basis for detection probability calculations [78]. To increase the detectability of an animal in an aerial survey, a camera system is used to simultaneously image the entire strip observed by the rear-seat observers. For instance, an aerial survey by rear-seat observers in Tsavo NP did not detect 60% of giraffes [79]. As the increased time devoted to processing data is considered to be the main disadvantage of the CMR method, the use of machine learning tools for automatic image recognition will become increasingly valuable, e.g., [80].

Knowledge of individual animals is additionally helpful in terms of genetic population management, including minimizing the mean kinship and the long-term effects of genetic diversity [81,82]. Individual identification provides an excellent opportunity for participatory (citizen) science projects to engage guides, tourists, and local communities, which, besides the scientific value, contribute to increased awareness and an enhanced positive approach towards conservation actions [83]. The genetic health of the West African giraffe was first assessed in 2008 and found to be surprisingly healthy, despite the bottleneck experienced during the 1990s [25]. However, these results are consistent with many studies of ungulates which tend to cope relatively well with high levels of inbreeding, including captive populations, which are then able to adapt when released into the wild, such as Przewalski horse (*Equus ferus przewalskii*) [84], Scimitar-horned oryx (*Oryx dammah*) [85], and Arabian oryx (*Oryx leucoryx*) [86].

An integrated community-based program has and continues to be a crucial part of West African giraffe conservation in Niger. Although concerns were raised regarding long-term human–giraffe coexistence, especially after the closure of PURNKO [32,46], human–giraffe conflicts have remained relatively rare [41,44,45,51]. However, with ever-increasing giraffe, human [87], and livestock [88] populations, competition for space and resources will rise and shape the future conservation attitudes and actions of the local people. Niger’s conservation policy is more participatory than fortress-oriented, and the coexistence with giraffe brings both costs and benefits to local communities, e.g., gaining local support, community development benefits, and reducing management costs [89]. Access to conservation-related benefits can positively influence local attitudes [90]. However, local communities adopt participative approaches in different ways, and if benefits are perceived as (increasingly) small in relation to losses, they may not produce the required effect. For example, in Tanzania (Selous Game Reserve), the benefits are seen as minimal when compared with costs [91]. Wildlife can endanger human lives as well as destroy crops, negatively influencing people’s attitudes towards conservation. Thus, this can be viewed as a constraint and a burden rather than as a development benefit [91]. In Botswana, rural communities showed negative attitudes towards conservation despite receiving substantial benefits from the licensed hunting of wildlife. This negative perception was individual rather than community-wide, caused by wildlife crop damages, loss of livestock to predators, loss of land to conservation, and lack of control over wildlife resources [92]. In Niger, the positive value of giraffe for local communities was also challenged by some local community members, particularly as they destroyed individual farmer crops. And the vision of them being a national heritage does not seem to be shared by all [93]. Awareness programs linked to giraffe conservation have aided local communities to value giraffes in terms of the benefits brought through development programs and ecotourism, but what will happen now that tourism is non-existent because of insecurity and the world COVID-19 pandemic? As one of the world’s poorest countries, such issues are a real threat to the long-term sustainability of the West African giraffe.

Environmental shifts resulting from habitat loss, degradation and fragmentation, and climate change may in future be exacerbated by increasing human-related disturbances and stochastic events, limiting giraffe seasonal movements and the use of current key forage areas in Niger. Migratory herbivores adapt their behavior to cope with such changes and remain synchronized with the peak of food availability in the landscape whilst minimizing the potentially negative effects on reproductive success [57]. However, the growing human population and the destruction of habitat will likely affect the West African giraffe. As the population increases, we can expect giraffe splitting into sub-populations and broader ranging behavior in search of suitable forage and fewer human disturbances. As such, they will potentially be at greater risk to poaching in neighboring countries which have not had the same level of awareness and benefits [17].

Understanding the movements and habitat use of threatened species is essential to effective conservation planning and management. Wildlife tracking technology has increased our capacity to collect and analyze vast datasets [94]. The knowledge obtained is critical, from the creation/maintenance of corridors for connectivity [95] to the protection of key habitats [96] and managing isolated populations [30]. Such technological advances will bring about more detailed insights into the monitoring of West African giraffe spatial behavior, as well as understanding their ecological needs and guiding effective long-term conservation in this ever-changing human-dominated landscape [30,97,98,99].

A key step in the long-term conservation of West African giraffe is their reintroduction to GBR. The establishment of new satellite populations will in time lead to the improved viability of the taxon and lower the negative effect of potential stochastic events. Prolonged drought impacted large areas of the Sahel in the 1970s and 1980s [60,100,101]; although, fortunately, the dry conditions have largely been reversed by increased rainfall in the early 2000s, leading to the ‘regreening of the Sahel’ [102,103,104]. In the context of climate change, the long-term trends in Niger show an average annual temperature increase of 0.21 °C and total rainfall increase by 26.3 mm per decade, despite the rainy days decreasing by 2.1 days/decade between 1979 and 2015 [105]. Since 2013, Niger´s Wildlife Authority, with support from the UNDP Niger Fauna Corridor Project, has worked diligently towards restoring the GBR habitat. From a giraffe perspective, the southern boundary of the reserve touches the northern limit of agricultural land, which may in time result in human–giraffe conflict [67]; although, to date, this has not been observed. In the long-term, re-introductions of the West African giraffe to other sites in Niger (and regionally) will be valuable for their conservation. Although post-release monitoring is recommended [66], it is often not undertaken, and results are rarely published. One of the potential indicators of giraffe translocation success is the establishment of movement patterns and HR in the new location [97]. In Namibia, four out of six translocated Angolan giraffe established HRs, while two exhibited long-distance movements [97]. Ongoing monitoring of the giraffe translocated to GBR continues, and despite an initial long-distance (>100 km) movement undertaken by four individuals, five years post-reintroduction, the initial (and subsequent) giraffes are very much resident in and around the reserve ([72], R. Zabeirou pers. comm.).

Moving forward, to best monitor the expanding West African giraffe population, we need to focus on new and/or adapted techniques for surveying and analysis, especially regarding gaining a higher reliability of population size estimates and/or trends. In terms of community conservation, increased efforts should be targeted to minimize human–giraffe conflict in both the ‘Giraffe Zone’ and GBR. Findings from targeted human dimension studies should be applied, and where appropriate, community-based incentives and livelihood opportunities should be further developed and linked to giraffe conservation efforts. The ongoing scientific interrogation of giraffe spatial movements and use will provide additional understanding of the mechanisms to cope with ecological and human-induced constraints. Additionally, the analyzed and modeled results can advise predictions for future reintroduced populations and the designing of (or maintaining) wildlife corridors and connectivity. Finally, the initial success of the GBR translocations serves as a flagship restoration program for large mammal populations across West Africa. In time, it is hoped that similar targeted efforts will be rolled out to secure other wildlife and habitats across the region.

## 5. Conclusions

To ensure the long-term coexistence of West African giraffe and local communities in Niger, the following is recommended: (1) a review of the current survey and monitoring techniques to help better monitor increasing population numbers and range expansion; (2) detailed analysis of human–giraffe conflicts, especially spatial crop-raiding assessment to target community-based incentives (consolation program) in highly populated and expansion areas; (3) development of habitat use and landscape connectivity models to determine key resource use and availability for giraffe and predicting the future expansion of their range; and (4) further translocations to augment current and (re)establish a suitable historical giraffe range.

## Data Availability

Datasets analyzed during the current study are available from the corresponding author.
